# Malaria time series in the extra-Amazon region of Brazil: epidemiological scenario and a two-year prediction model

**DOI:** 10.1186/s12936-022-04162-1

**Published:** 2022-05-31

**Authors:** Klauss Kleydmann Sabino Garcia, Amanda Amaral Abrahão, Ana Flávia de Morais Oliveira, Karina Medeiros de Deus Henriques, Anielle de Pina-Costa, André Machado Siqueira, Walter Massa Ramalho

**Affiliations:** 1 Tropical Medicine Center, University of Brasilia, Darcy Ribeiro University Campus, Brasília, Brazil; 2 Health Ministry of Brazil, Federal District, Brasilia, Brazil; 3 Federal Institute of Education, Science and Technology of Tocantins, IFTO, Araguaína, Brazil; 4 FIOCRUZ, Evandro Chagas National Institute of Infectious Diseases, Av. Brasil, Rio de Janeiro, RJ 4365 Brazil; 5 School of Medicine, Centro Universitário Serra Dos Órgãos (UNIFESO), Teresópolis, RJ Brazil

**Keywords:** Malaria, Malaria Case, Epidemiology, Public Health, Control; Elimination, Extra-Amazon

## Abstract

**Background:**

In Brazil, malaria is caused mainly by the *Plasmodium vivax* and *Plasmodium falciparum* species. Its transmission occurs in endemic and non-endemic areas. Malaria geography in Brazil has retracted and is now concentrated in the North region. The Brazilian Amazon region accounts for 99% of Brazil's cases. Brazil’s extra-Amazon region has a high frequency of imported cases and in 2019 presented a mortality rate 123 times higher than the Amazon region. Extra-Amazon cases present risks of reintroduction. This study aims to characterize the epidemiological scenario for malaria in the extra-Amazon region of Brazil from 2011 to 2020 with a two-year forecast.

**Methods:**

Time-series study with description of malaria cases and deaths registered in Brazilian extra-Amazon region from 2011 to 2020. Public data from the Notifiable Diseases Information System (Sinan) and the Mortality Information System (SIM) were used. Descriptive analysis, incidence, and notification rates were calculated. Flow charts analysed the flux between Places of Probable Infection (PI) and places of notification. The prediction model utilized a multiplicative Holt-winters model for trend and seasonality components.

**Results:**

A total of 6849 cases were registered. Cases were predominantly white males with 9 to 11 years of education, mostly between 30 and 39 years old. Imported cases accounted for 78.9% of cases. Most frequent occupations for imported cases are related to travelling and tourism activities. Among autochthonous cases, there is a higher frequency of agriculture and domestic economic activities. In the period there were 118 deaths due to malaria, of which 34.7% were caused by *P. falciparum* infections and 48.3% were not specified. The most intense flows of imported cases are from Amazonas and Rondônia to São Paulo, Rio de Janeiro, and Paraná. The prediction estimates around 611 cases for each of the following two years.

**Conclusion:**

The time series allows a vast epidemiological visualization with a short-term prediction analysis that supports public health planning. Government actions need to be better directed in the extra-Amazon region so the objective of eliminating malaria in Brazil is achieved. Carrying out quality assessments for information systems and qualifying personnel is advisable. Malaria outside the Amazon region is mainly due to imported cases and delay in diagnosis is associated with a higher fatality rate. Better strategies to diagnose and treat suspected cases can lead to lower risk of deaths and local outbreaks that will be important for achieving malaria elimination in Brazil.

**Supplementary Information:**

The online version contains supplementary material available at 10.1186/s12936-022-04162-1.

## Background

Malaria is an acute febrile infectious disease, caused by a protozoan of the genus *Plasmodium* that infects humans through female *Anopheles* mosquito bites. It is common in tropical and developing countries and is considered a neglected tropical disease by the World Health Organization (WHO). Malaria affects about 212 million people worldwide and caused more than 409,000 deaths in 2019, with mortality rates ranging from 0.2 to 2.2 worldwide [1, 2].

In Brazil, the main *Plasmodium* species are *Plasmodium vivax* and *Plasmodium falciparum*, but there is also the transmission of *Plasmodium malariae*, although its occurrence is low compared to that of *P. vivax* and *P. falciparum*. Transmission by *P. ovale* has not been reported in Brazil in previous literature. Malaria transmission in endemic and non-endemic areas relates to the particular *Anopheles* vector species, common in areas of the Amazon Forest and the Atlantic Forest [3–6].

Historically, malaria in Brazil has undergone changes in its geographic distribution. Its occurrence during the twentieth century was prevalent throughout the country but in the last 50 years it has become concentrated in the Brazilian Amazon region [7, 8]. The Legal Amazon region comprises approximately 99% of all cases in the country. Although the extra-Amazon region accounts for only 1% of the total cases, it is noteworthy that cases in this region presents higher lethality and represent a possible risk for a re-establishment of malaria transmission [9]. From 2011 to 2020, Brazil registered about 1.8 million cases of malaria with 450 deaths [10].

Although the total number of deaths from malaria in the extra-Amazon region is lower than in the legal Amazon region, the relative case fatality was 123 times greater than in the Amazon region in 2019 [3, 11]. Thus, this study aims to describe and characterize the malaria epidemiological scenario and profile in the extra-Amazon region of Brazil over the last 10 years.

## Methods

### Study design

Ecological time-series study with the description of reported cases and deaths from malaria in the Brazil extra-Amazon region from 2011 to 2020. In this study, the following definitions for malaria cases were adopted: Reported case: Case with a positive laboratory result for malaria; new infection (new case): Case with positive laboratory result for malaria excluding positive Cure Slide Verification (CSV); Autochthonous cases: Classification based on the place of probable infection (municipality or state). Negative cases were not analysed in this study. Malaria deaths were analysed according to the place of residence of the case [12, 13].

### Study site

The study site selected was the Brazilian extra-Amazon region, which includes 17 states (Piauí, Ceará, Rio Grande do Norte, Paraíba, Pernambuco, Alagoas, Sergipe, Bahia, Minas Gerais, Espírito Santo, Rio de Janeiro, São Paulo, Paraná, Santa Catarina, Rio Grande do Sul, Mato Grosso do Sul, Goiás) and the Federal District. It is noteworthy that the Legal Amazon region covers all the states of the North region plus the states of Maranhão, Mato Grosso, and Tocatins[14]. According to the Brazilian Institute of Geography and Statistics (IBGE) population projections, the extra-Amazon region hadan average of 176,163,089 inhabitants, per year between 2011 and 2020.

### Data source and analysis

Public data from the Notifiable Diseases Information System (Sinan) were obtained from the Brazilian Ministry of Health (MoH) database portal – DATASUS – in May 2021. MoH provided Mortality data (SIM) through the Integrated Ombudsman and Access to Information Platform of the Brazilian Federal Government in June 2021. For data processing and analysis the following software were used: Qgis Desktop (2.18), Microsoft Excel (2016) and the R software packages read.dbc, tidyverse, lubridate, forecast, and fpp (version 4.0.5) [15].

The analysed variables were: Notification date, Symptom onset date, Treatment start date, Birthdate, Age, Gender, Race/Color, Education, Pregnant woman, Activity in the last 15 days, professional Activities, Type of slide (active, passive detection, CSV), Examination results, Autochthony, Notification locations – Municipality and State (denoted as FU—Federated Units), PI and death location.

### Incidence rates

Malaria incidence rates (total new malaria cases by the PI municipality /Total population of the municipality*100,000) and malaria notification rates (total malaria cases by the notification municipality /Total population of the municipality *100,000) were calculated for each year. Incidence rates calculated by the PI consider that those cases are autochthonous. The total new infections number is a result of the total of positive cases in the period excluding those classified as CSV,which represent recurrences of the infection [12].

The Qgis software plugin "Flowmaps" (version 2.18) was used to analyse flows between PI and the place of notification of new malaria infections. Along with the flow analysis the average incidence rate for the period was described (Average of: Total new infections number by the PI municipality for each year/ Resident population by the municipality for each year × 100,000 inhabitants).

The R software commands ts, decompose, Holt-winters, and forecast were used to predict cases for 2021 and 2022. For the prediction analysis, the attributes of mean, trend, and seasonality of notified malaria cases from 2011 to 2020 were considered. Holt-winters exponential smoothing and prediction methods with a multiplicative seasonality influence in modelling was used, as in the study of Swapnarekha et al. [16]. The Holt-winters seasonal multiplicative model multiplies the series trend by the seasonality, instead of adding one to the other, as is it in the Holt-winters additive modelling. The multiplicative model is better suited to malaria’s time series because its seasonality has a higher influence on increasing or decreasing cases number. If the seasonality did not have such a high influence on the time series, the additive model would be a better choice to do the forecast modelling.

The Holt-Winters prediction model evaluates the time series behavior based on the patterns of the three attributes mentioned earlier. Exponential smoothing uses an exponentially weighted moving average (EWMA) to level the time series and provide a higher quality forecast. This model uses exponential smoothing to consider historical values and predicts a likely future value considering 95% confidence intervals [17].

This study did not require approval by ethics committees according to the Brazilian National Health Council resolution nº 466/2012. This work received funding from the Bill & Melinda Gates Foundation (INV-003970) and the Brazilian Ministry of Health (MoH/DECIT/CNPq #443,148/2019–8). All data generated or analysed during this study are included in this published article Additional files 1, 2 and 3.

## Results

From 2011 to 2020, the Brazilian extra-Amazon region registered 6,849 notifications of confirmed malaria cases, of which were 682 (9.9%) cases classified as CSV, and 6,167 (90.1%) were new malaria cases notifications, where 1,640 (26.5%) were notified through active detection.

Cases were predominantly male (78%), white (44.3%), and mixed (38.7%); the distribution by race/colour is similar between males and females. The highest concentration by age group is in 20 to 59 years (84%). The age group 30 to 39 years presents the highest frequency of cases (26.1%), along with white or brown people (42.8% and 38%, respectively). Among the positive cases, 58 cases were found to have a self-reported age of less than one year, with the median being 37 years (1-quartile: 28; 3-quartile: 49; maximum: 93). As for education, the highest frequencies are in people with 9 to 11 years of schooling (21.7%). For “Education level”, 23.6% were filled out as “ignored” (Table 1).

**Table 1:** Epidemiological profile of malaria cases in the extra-Amazon region of Brazil, 2011 to 2020

Sex						
	Male		Women		Total	
	N	%	n	%	N	%
Total	5343	78.01	1506	21.99	6849	100.00
Age groups						
Under 1 year old	45	0.84	14	0.93	59	0.86
01—04	52	0.97	29	1.93	81	1.18
05—09	37	0.69	24	1.59	61	0.89
10—14	85	1.59	54	3.59	139	2.03
15—19	180	3.37	69	4.58	249	3.64
20—29	1096	20.51	357	23.71	1453	21.21
30—39	1461	27.34	324	21.51	1785	26.06
40—49	1116	20.89	265	17.60	1381	20.16
50—59	817	15.29	227	15.07	1044	15.24
60—69	353	6.61	87	5.78	440	6.42
70—79	87	1.63	45	2.99	132	1.93
80 years or more	14	0.26	11	0.73	25	0.37
Total	5343	100	1506	100	6849	100.00
Race/Color						
White	2319	44.35	610	41.16	2,929	43.64
Mixed	2025	38.73	578	39.00	2,603	38.79
Black	470	8.99	158	10.66	628	9.36
Indigenous	17	0.33	16	1.08	33	0.49
Yellow	35	0.67	14	0.94	49	0.73
Ignored	363	6.94	106	7.15	469	6.99
Total	5229	100	1482	100	6711	100.00
Years of study						
None	49	1.0	13	0.9	62	1.0
1 to 4	508	10.4	131	9.4	639	10.2
5 to 8	913	18.7	196	14.1	1109	17.7
9 to 11	1215	24.9	276	19.8	1491	23.8
12 or more	861	17.6	341	24.5	1202	19.2
Not applicable	106	2.2	50	3.6	156	2.5
Ignored	1230.00	25.2	387	27.8	1617.00	25.8
Total	4882	100	1394	100	6276	100
Professional activities						
Traveller	931	19.03	303	20.21	1,23	19.31
Tourism	447	9.14	245	16.34	692	10.83
Agriculture	457	9.34	89	5.94	546	8.54
Panning	443	9.05	40	2.67	483	7.56
Constructor	239	4.88	3	0.20	242	3.79
Mining	192	3.92	39	2.60	231	3.61
Domestic	52	1.06	178	11.87	230	3.60
Hunting/ Fishing	108	2.21	6	0.40	114	1.78
Livestock	68	1.39	5	0.33	73	1.14
Expl. vegetable	57	1.16	5	0.33	62	0.97
Others	141	28.88	397	26.48	181	28.32
Ignored	486	9.93	189	12.61	675	10.56
Total	489	100	15	100	639	100.00

Source: Sinan—Brazilian Ministry of Health

As for occupations, the most frequent activities are related to “travel” and “tourism”, which together account for 31.2% of the total number of records for malaria cases in the extra-Amazon region. Women have a higher relative frequency of activities related to "tourism" and "domestic" employees, whereas men have a higher relative frequency of cases in "agriculture", "construction" and "mining" activities (Table 1). Analysing the occupations according to the level of education, the most frequent are “Traveller” and “Tourism”, which account for 4.6% and 2.6% when over 12 years of study, respectively.

For autochthonous cases, the most frequent activities are “Agriculture” (264 cases; 26.9%) and “Domestic” (93 cases; 9.5%). Imported cases are more frequent in “Travellers” (1,110 cases; 22.2%) and “Tourism” (630 cases; 12.5%). Information filled as “Other” economical activities account for a total of 25.8% of autochthonous cases and 28,4% of imported cases (Table 2). Information about occupational activities in death notifications shows that the most frequent deaths in the extra-Amazon region are of retired people (16 deaths; 13.7%), housewives (6 deaths; 5.1%) and, managers in commercial activities (6 deaths; 5.1%).

**Table 2:** Cases distribution by *Plasmodium* species, professional activity, and autochthony, 2011 to 2020, Brazil’s extra-Amazon region

	Autochthonous		Imported		Undetermined		Total	
	n	%	n	%	n	%	n	%
Type of malaria for new infections								
Falciparum	212	21.1	1805	33.4	96	31.3	2113	31.4
Vivax	785	78.0	3539	65.4	200	65.1	4524	67.3
Ovale	2	0.2	42	0.8	5	1.6	49	0.7
Malariae	8	0.8	25	0.5	6	2.0	39	0.6
Total	1007	100	5411	100	307	100	6725	100
Economic activities								
Traveller	51	5.2	111	22.2	62	21.4	1223	19.5
Tourism	40	4.1	630	12.6	17	5.9	687	10.9
Agriculture	264	26.9	252	5.0	16	5.5	532	8.5
Pannig	37	3.8	420	8.4	13	4.5	470	7.5
Constructor	19	1.9	215	4.3	4	1.4	238	3.8
Domestic	93	9.5	127	2.5	7	2.4	227	3.6
Mining	25	2.5	180	3.6	21	7.2	226	3.6
Hunting/Fishing	15	1.5	95	1.9	3	1.0	113	1.8
Livestock	16	1.6	52	1.0	2	0.7	70	1.1
Vegetal exploration	22	2.2	40	0.8		–	62	1.0
Others	253	25.8	1423	28.4	96	33.1	1772	28.2
Ignored	146	14.9	462	9.2	49	16.9	657	10.5
Total	981	100	5006	100	290	100	6277	100

Source: Sinan—Brazilian Ministry of Health

Among the new malaria cases in the period, 912 (15%) records were notified as autochthonous cases, 4,869 (78.9%) as imported and 307 (4.5%) were indeterminate. Malaria due to *P. vivax* accounted for 4,592 cases (67.5%) and *P. falciparum* or mixed malaria cases (*P. falciparum* malaria along with other species) accounted for 2,159 (31.5%) notifications. There is also a total of 39 malaria cases caused by *P. malariae* and 59 cases caused by *P. ovale*, which represent only 1.4% of the total number of confirmed cases over the time series (Table 2).

**Fig. 1:**
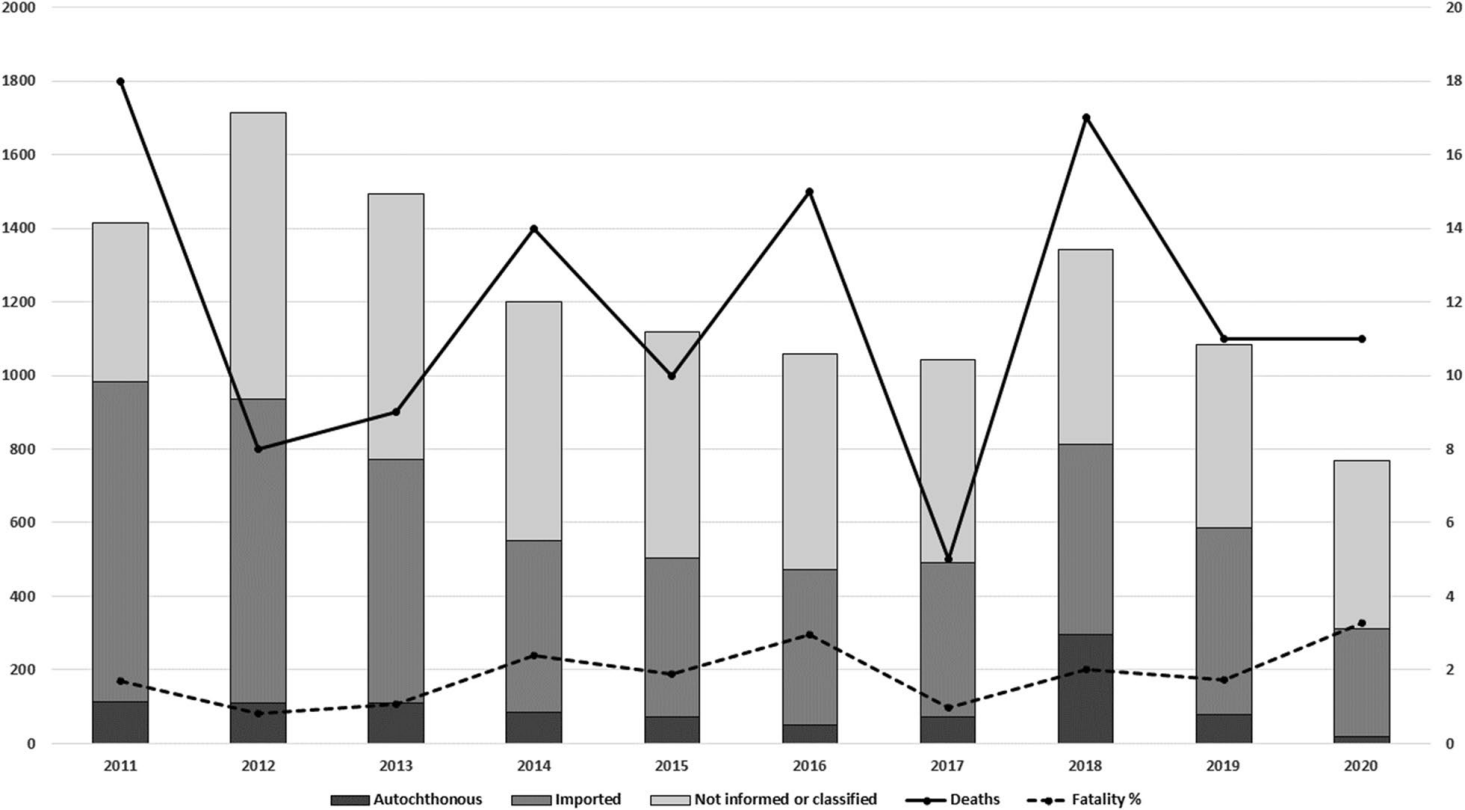
Cases, deaths, and fatality rate from malaria in Brazil’s extra-Amazon region, 2011 to 2020. Source: Sinan and SIM – Brazilian Ministry of Health

The time series presented an approximate average of 685 cases per year, with the highest number of cases reported in 2011, at 1,066 cases, with the lowest being 336 cases in 2020. The monthly average was 57 cases (minimum of 10 and maximum of 177). From 2011 to 2020, there were 118 deaths, with a minimum of five deaths in 2017 and a maximum of 18 in 2011 (Fig. 1). Of these, 41 (34.7%) deaths were due to *P. falciparum*, 17 (14.4%) due to *P. vivax*, 3 (2.5%) due to *P. malariae*, and 56 (48.3%) due tounspecified malaria.

FU most frequent autochthonous cases occurred in Espírito Santo (411 cases; 6%), Bahia (136 cases; 2%) and São Paulo (123 cases; 1.8%). The states that most reported malaria cases were São Paulo (1,405 reported cases; 20.5%), Rio de Janeiro (778; 11.4%), and Minas Gerais (741; 10.8%). For imported cases, most cases are notified in São Paulo (1,214 cases; 22.4%), Minas Gerais (691 cases; 12.8%), and Rio de Janeiro (649 cases; 12%).

**Fig. 2:**
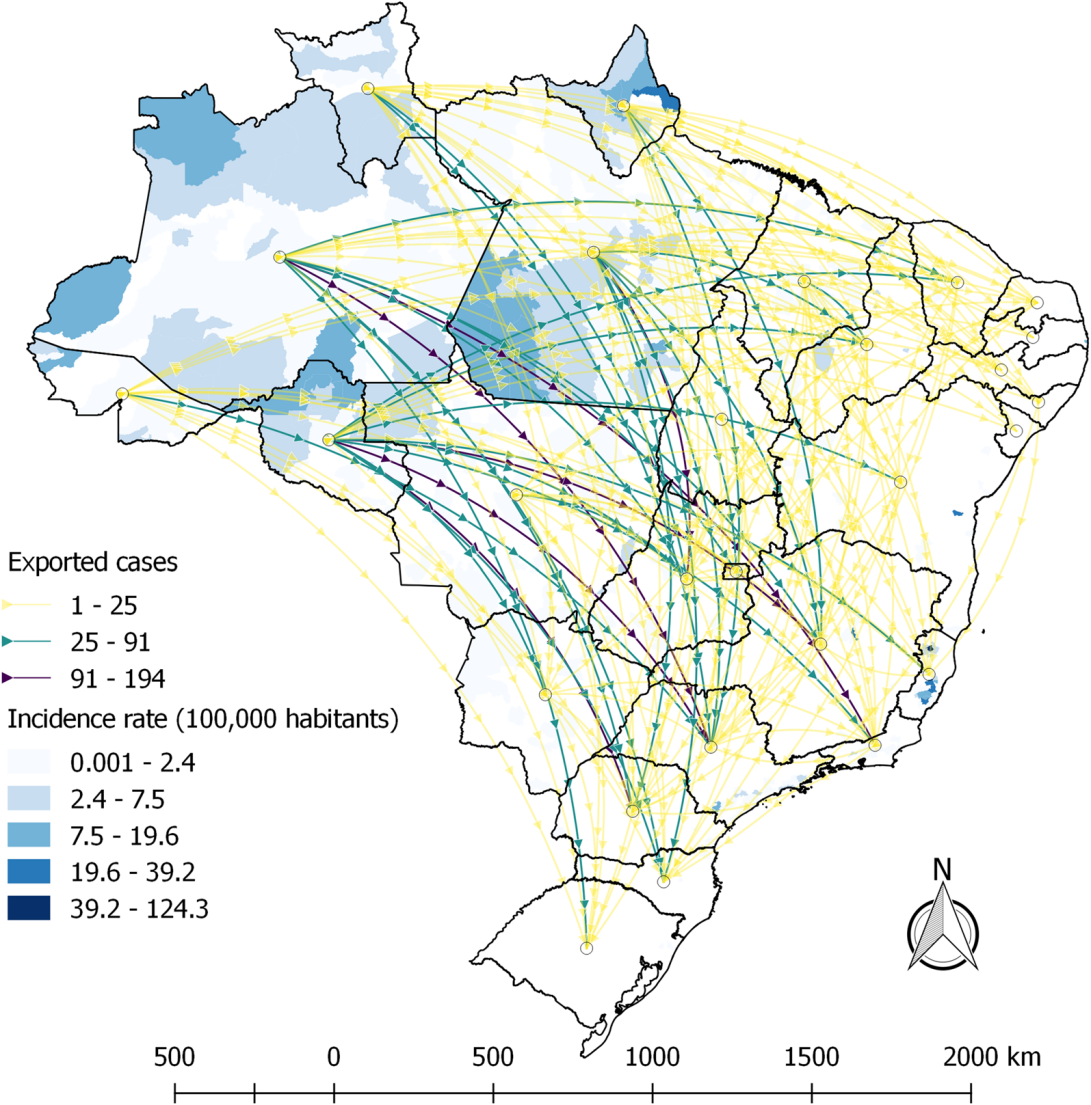
Malaria cases flow with infection location in-country notified in Brazil’s extra-Amazon region with incidence rates. 2011 to 2020. Source: Sinan—Brazilian Ministry of Health

Of the 118 deaths in the extra-Amazon region, they were mostly of residents from São Paulo (30 deaths; 25.4%), Minas Gerais (21 deaths; 17.8%), Bahia, and Goiás (9 deaths each; 7.6%). Figure 2 shows that the most intense flows were in the North region, mainly the states of Amazonas and Rondônia to the states of São Paulo, Rio de Janeiro, and Paraná (Fig. 2).

Annual incidence rates for the entire region ranged from 0.16 to 0.60 cases per 100,000 inhabitants, whereas municipal incidence rates ranged from 0.008 (São Paulo/SP in 2020) to 1,243 (Vila Pavão/ES in 2018) cases for every 100,000 inhabitants (average: 11—median: 4.6). The notification rates showed a similar annual variation, ranging from 0.03 (Salvador/BA in 2020) to 1,286.6 (Vila Pavão/ES in 2018) cases reported for every 100,000 inhabitants (mean 6.7—median: 1.6). The extra-Amazon malaria fatality rate from 2011 to 2020 was 1.8, with a minimum of 0.81 in 2012 and a maximum of 3.27 in 2020 (Fig. 1).

As for other imported cases, Brazil notified 1,771 new infections from other countries in the time series. The countries responsible for up to 80% of the total imported cases are Angola (534–30.2%), South Africa (163–9.2%), Mozambique (158–8.9%), French Guiana (137–7.7%), Guyana (132–7.5%), Nigeria (124–7.0%), Venezuela (116–6.5%) and Equatorial Guinea (60–3.4%).

**Fig. 3:**
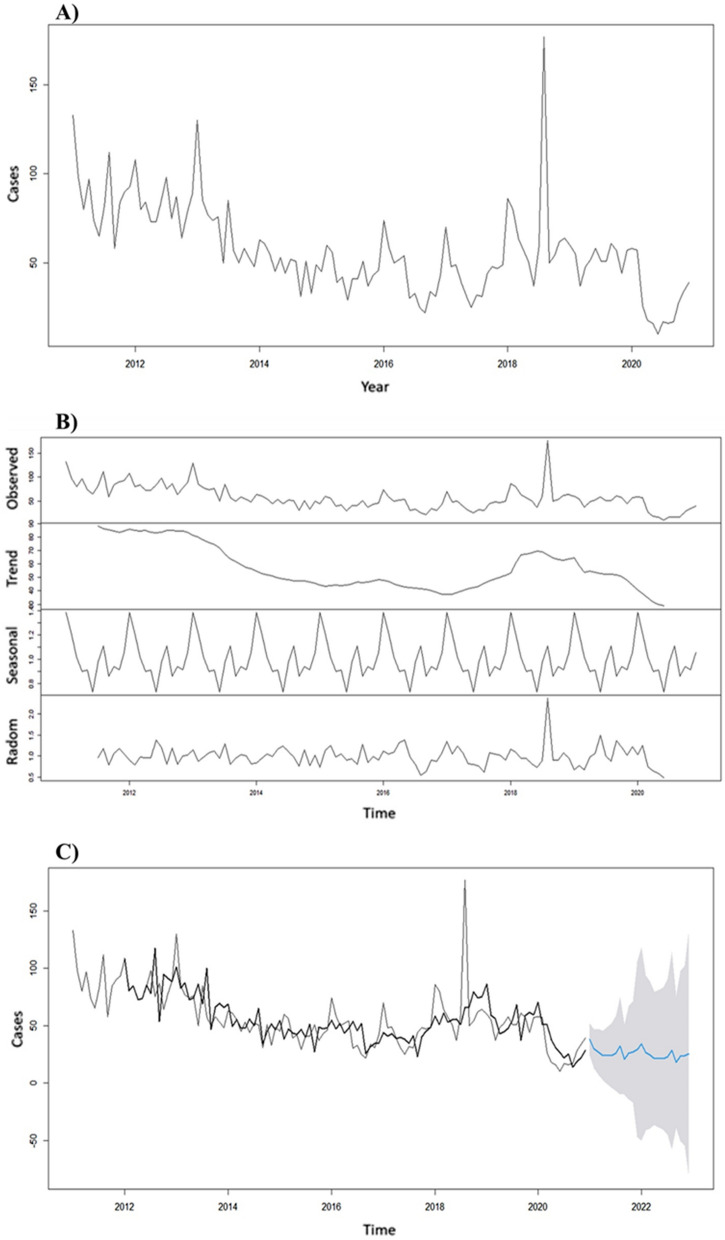
Brazilian extra-Amazon region Malaria time series, trend, seasonality, and randomness attributes with two-year prediction. Source: Sinan—Brazilian Ministry of Health. **A** Brazilian extra-Amazon region malaria time series; **B** Malaria time series components; **C** Malaria cases prediction for 2021 and 2022

The prediction model used presented estimates for the months of 2021 and 2022, with an average of 27 cases per month in the first year and 24 monthly cases in 2022. The time series showed a cases reduction trend. The estimates are accompanied by confidence intervals that, when predicted for periods longer than 12 months start to lose precision in their estimates, thus, it is noteworthy that for the year 2021, 324 cases were predicted with a minimum of 16 and a maximum of 47 cases in a single month (Fig. 3). 611 cases were predicted for the following two years.

## Discussion

Considering that Brazil has a goal of eliminating malaria cases by the year 2035 [11] new malaria cases and recurrences were analysed together in the epidemiological profile, even though the database recurrences number are probably underestimated. From the perspective of elimination, recurrence cases must be considered in the time series analysis of the profile, because an untreated recurrence case not treated may become an outbreak source, and it needs to receive malaria treatment. As for the predictive analysis, only new malaria cases were used because 79% of the cases notified in the extra-Amazon region were imported new infections from the Amazon region—as in the study of Machado and collaborators [18]. It is important to say that CSV is underestimated in Sinan because imported cases could move back to their regions and so it is notified in a different information system or another country.

The results highlight that the occurrence of malaria cases in men, as it is in the Amazon region, is higher than in women. Additionally, the cases are made up of mostly adults (20 to 59 years) and have a similar distribution between people of white and brown color/race. The distribution of malaria cases by age group, for the same period, differs from the profile of cases reported in the Amazon region, where the concentration of cases in people under 20 years of age is approximately 46.3% of the total cases. In the extra-Amazon region, the portion of people under 20 years of age does not exceed 9%. As for the distribution of cases by race/colour, the profile is also different because in the Amazon region about 60.8% of cases are brown, 17.6% indigenous and only 6.2% are white (data updated on 06–09-2021) [19, 20].

Considering that most cases reported in the extra-Amazon region are imported cases and that travelling to endemic areas is a known risk factor for contracting malaria [21], this difference in the profile may be related to socio-economic issues such as years of schooling, economic activity, and income. This indicates that the occurrence of malaria cases in the extra-Amazon and Amazon regions of Brazil relates to social inequalities.

The MoH traditionally presents different strategies for both regions, however, it is suggested that specific internal control goals for the country be worked out for the extra-Amazon region [11, 12, 19].

The notification of autochthonous cases in the extra-Amazon region is a warning to epidemiological surveillance centers as it represents the establishment of community transmission, which is a risk for the reintroduction of malaria in these non-endemic, but still susceptible regions [22]. It is important to highlight that approximately 27.5% of the extra-Amazon cases were notified in the year 2018, which was an atypical year for malaria control in Brazil, as the country was showing constant reductions in malaria rates until 2017. In 2016 Brazil had reported approximately 129,000 cases and in 2017 reported over 194,000 [11].

When analysing the PI and the notification place in the extra-Amazon region according to their professional activity, it became clear that a large number of imported malaria cases are from people with professional activities related to travelling or tourism, with the mining activities also standing out. There is a portion of cases that were classified as indeterminate autochthony which are patients with professional activity also related to travelling [23, 24]. Agriculture activities stand out as the most frequent activity, being the most frequent between autochthonous cases. This may be related to the area that they work or live in, considering that these activities may be agriculture for self-sustaining. The high number of cases with undetermined PI also stood out. It should be noted that epidemiological information is as important as the response to possible outbreaks. It is advisable that specific studies be carried out to determine job-related malaria characteristics in Brazil, given that over 38% of economic activities are blank, ignored, or defined as “Other”.

The data has shown two autochthonous cases of malaria by *Plasmodium ovale* in Brazil, which is a parasitic species with no previous records of community transmission in the country [3, 22, 25]. These notifications should be reviewed by the state and municipal health departments to better understand these cases and certify whether or not there was community transmission. These notifications may be system-filling or diagnostic errors because they were isolated cases, one in 2011 infected in the state of Rondônia and reported in Rio de Janeiro and another in 2014 infected and reported in Espírito Santo.

It is important to highlight that 4.4% of the records for the period had no defined autochthony. Therefore, epidemiological investigation in the extra-Amazon region needs improvement.

As long as the Amazon region presents a high incidence of autochthonous malaria cases the extra-Amazon case notifications will not decrease. It is necessary to understand that the occurrence of autochthonous and imported cases in the extra-Amazon region follows a seasonal pattern and a trend. Those components’ behaviour is related to the malaria epidemiological behavior in the Amazon region [18, 23].

It is critical that specific actions and policies for preventing deaths be directed to the extra-Amazon region, mainly in the states of São Paulo, Minas Gerais, and Goiás, which are the ones with the highest accumulated number of deaths in the last 10 years. Those states may have the highest number of deaths because there have the largest fluxes of travellers and people exposed to infections. Therefore, they are the states with highest number of cases and the number of deaths and fatality rate reflects failures in the identification and opportune treatment of cases. Policies must be directed at identifying suspected cases, testing them and providing proper care. International studies highlight some common risk factors for the occurrence of deaths, such as cases of non-autochthonous malaria, or that come from endemic regions, or that experience therapeutic failures or treatment delay [26, 27].

As shown by Duarte and collaborators [7] it is necessary to implement control and prevention strategies tailored to the realities of each geographic grouping. In addition, it is necessary to assess the quality of death notifications data, as the highest frequency of deaths was for ICD-10 B54 which is "Malaria not specified". The literature [22], as well as results from this study, suggests a high frequency of deaths from *P. falciparum* in the extra-Amazon region. Linkages techniques should be used in official health information systems for better specification of the species related to malaria deaths.

According to the Brazilian Health Ministry online data, the predictions for the 2021 initial 3 months were close to the value registered in Sinan for malaria cases in the extra-Amazon region (predicted: 108; observed: 113) [20]. Thus, the developed prediction allows better planning of specific control strategies for the containment of malaria cases, especially when considering the seasonality component of the disease [23, 28, 29]. The trend towards a reduction in cases in the extra-Amazon region can be interpreted as a result of the success in disease control and prevention actions carried out on the national and local levels. The model used in this study can be replicated on a state level.

The decrease in 2020 may relate to travel restrictions in the face of the COVID-19 pandemic, thus reducing visits to endemic areas in the North region of the country. This behaviour was previously noted in notifiable diseases in Australia and Taiwan, and this may also have occurred in Brazil [30, 31].

As for the limitations of this study, there is the fact that secondary data were used; the database had some missing information, which is shown by the results tabulations values that do not match each other. This difference is because some notifications do not have all the information on their investigation form filled out. In addition, defining autochthony of death is not possible due to SIM databank limitations. Linkage techniques may be an interesting strategy to present additional and more accurate information. Lastly, occupational activities in the SIM database may not be precise; therefore, further investigation is advised.

## Conclusions

A preventive and early detection measure that could be tested for screening malaria cases is to use rapid tests in bus stations and airports to test travellers presenting symptoms or those very high-risk areas for malaria. Another possible measure is, distributing an information booklet on malaria in these places with the description of the disease and guidance to the health professional on how to act when detecting the main symptoms.

The analysis of time series allows for a better understanding of its epidemiological behavior and, when accompanied by short-term predictions, can serve as tools to support government decision-making.

To achieve malaria elimination the government must invest in qualifying personnel that fill out malaria notifications and diagnose malaria, while also carrying out quality assessments of the Sinan and SIM databases.

Thus, one step to achieve the national elimination goal is the elimination of malaria in the extra-Amazon region of Brazil, preventing cases and deaths from the disease. Although malaria outside the Amazon represents a relatively limited portion of cases, it still accounts for a considerable number of cases and deaths, and also poses a risk for reintroduction illustrated by several local transmission outbreaks. To eliminate malaria from the country it is essential that a more timely and comprehensive surveillance is established in order to reduce these threats through better provision of care to suspected cases.

## Supplementary Information

**Additional file 1.** A script for software R containing programming used to develop the analysis.

**Additional file 2.** The study database containing malaria cases in brazil’s extra-amazon region for the analyzed period.

**Additional file 3.** The study database containing malaria deaths in brazil’s extra-amazon region for the analyzed period.

## Data Availability

The datasets generated and/or analysed during the current study are available in the OneDrive repository [https://saudegov-my.sharepoint.com/:f:/g/personal/klauss_garcia_saude_gov_br/EvBGbHi8SpxEoHEYtu7hw1wBFFj3dyZQUPqsmdln_91cUA?e=IJIPAT] and in the public website of MoH (DATASUS), link: https://datasus.saude.gov.br/transferencia-de-arquivos/.

## Abbreviations

MoH: Ministry of Health
CSV: Cure Slide Verification
FU: Federated Units
ICD: International Classification of Diseases
PI: Place of Probable Infection
SIM: Mortality Information System
Sinan: Notifiable Diseases Information System
WHO: World Health Organization
